# Analysis of apolipoprotein E genetic polymorphism in a large ethnic Hakka population in southern China

**DOI:** 10.1590/1678-4685-GMB-2017-0301

**Published:** 2018-11-29

**Authors:** Zhixiong Zhong, Heming Wu, Hesen Wu, Pingsen Zhao

**Affiliations:** ^1^ Center for Precision Medicine, Meizhou People's Hospital (Huangtang Hospital), Meizhou Academy of Medical Sciences, Meizhou Hospital Affiliated to Sun Yat-sen University, Meizhou, P.R. China; ^2^ Center for Cardiovascular Diseases, Meizhou People's Hospital (Huangtang Hospital), Meizhou Academy of Medical Sciences, Meizhou Hospital Affiliated to Sun Yat-sen University, Meizhou, P.R. China; ^3^ Clinical Core Laboratory, Meizhou People's Hospital (Huangtang Hospital), Meizhou Academy of Medical Sciences, Meizhou Hospital Affiliated to Sun Yat-sen University, Meizhou, P.R. China.

**Keywords:** Apolipoprotein E, genetic polymorphism, Hakka, southern China, genotyping

## Abstract

There is currently no data about the genetic variations of *APOE* in Hakka population in China. The aim of this study was to analyze the allelic and genotypic frequencies of *APOE* gene polymorphisms in a large ethnic Hakka population in southern China. The *APOE* genes of 6,907 subjects were genotyped by the gene chip platform. The allele and genotype frequencies were analyzed. Results showed that the ∊3 allele had the greatest frequency (0.804) followed by ∊2 (0.102), and ∊4 (0.094), while genotype ∊3/∊3 accounted for 65.43% followed by ∊2/∊3 (15.85%), ∊3/∊4 (14.13%), ∊2/∊4 (3.01%), ∊4/∊4 (0.84%), and ∊2/∊2 (0.74%) in all subjects. The frequencies of the ∊4 allele in Chinese populations were lower than Mongolian and Javanese, while the frequencies of the ∊2 allele were higher and ∊4 allele lower than Japanese, Koreans, and Iranian compared with the geographically neighboring countries. The frequencies of ∊2 and ∊4 alleles in Hakka population were similar to the Vietnamese, Chinese-Shanghai, Chinese-Kunming Han and Chinese-Northeast, and French. The frequency of ∊2 in Hakka population was higher than Chinese-Dehong Dai and Chinese-Jinangsu Han. The low frequency of the *APOE* ∊4 allele may suggest a low genetic risk of Hakka population for cardiovascular disease, Alzheimer’s disease, and other diseases.

## Introduction

Apolipoprotein E (ApoE) is a multifunctional protein that plays an important role in lipoprotein metabolism, and is involved in the metabolism of very low density lipoproteins (VLDL) and chylomicrons (Blum, 2016). There are three major isoforms of human ApoE including E2 (OMIM 107741.0001), E3 (OMIM 107741.0015), and E4 (OMIM 107741.0016), as identified by isoelectric focusing. The gene coding for ApoE is *APOE* (OMIM 107741), which is located on chromosome 19 in band 19q13.32 (Mahley, 1988; Siest *et al.*, 1995). The polymorphisms in the fourth exon of *APOE* gene determine three common alleles (∊2, ∊3 and ∊4) coding for three major isoforms of ApoE (Martin *et al.*, 2000; Kantarci *et al.*, 2004; Kumar *et al.*, 2017).

The E2, E3, and E4 isoforms differ in amino acid sequence at two sites, residue 112 (called site A) and residue 158 (called site B). At sites A/B, ApoE2, ApoE3, and ApoE4 contain cysteine/cysteine, cysteine/arginine, and arginine/arginine, respectively, which are encoded by ∊2, ∊3, and ∊4, respectively (Weisgraber *et al.*, 1981; Rall Jr *et al.*, 1982a). By different combinations of these three alleles, six genotypes (∊2/∊2, ∊2/∊3, ∊2/∊4, ∊3/∊3, ∊3/∊4, and ∊4/∊4) are formed (Svobodová *et al.*, 2007b; Yousuf *et al.*, 2015). Some studies pointed out that the ∊3 allele is the most frequent in all human groups, while *APOE* ∊3/∊3 is the most common genotype in most population (Corbo and Scacchi, 1999; Al-Dabbagh *et al.*, 2009; Achourirassas *et al.*, 2016; Jairani *et al.*, 2016; Monge-Argilés *et al.*, 2016; Tanyanyiwa *et al.*, 2016).

Meizhou is a city covering the northeast of Guangdong Province, which connects to Fujian, Guangdong, and Jiangxi provinces, with an area of 15,876 km^2^ and a population of 5.44 million. The vast majority of the residents living in this area are Hakka. Hakka is an intriguing Han Chinese population that mainly inhabits southern China and that migrated south originally from the Reaches of Yellow River (Li, 1997). There is currently no data about the genetic variations of *APOE* gene in the Hakka population.

## Material and Methods

### Subjects

For this study, 6,907 Chinese Hakka subjects were included through February 2016 to August 2017. Subjects visited Meizhou *People’s Hospital (Huangtang Hospital), Meizhou* Hospital Affiliated to Sun Yat-sen University located in Guangdong province in China. The present study was performed in accordance with the ethical standards laid down in the updated version of the 1964 Declaration of Helsinki and approved by Human Ethics Committees of Meizhou *People’s Hospital*. All the patients had signed the informed consent.

### DNA extraction

Blood samples were stored in 2-mL vacuum tubes containing ethylenediaminetetraacetic acid (EDTA) from each participant. Genomic DNA was extracted from the samples using QIAamp DNA Blood Mini Kit (Qiagen, Germany) according to the manufacturer’s instructions. DNA concentration and purity were quantified using Nanodrop 2000^TM^ Spectrophotometer (ThermoFisher Scientific, Waltham, MA), and only good quality DNA (A260/280 ratio > 1.7) was stored at -80 °C up to the day of analysis.

### Polymerase chain reaction and genotyping

The single nucleotide polymorphisms of *APOE* gene rs429358 and rs7412 were genotyped using a commercially available kit (Sinochips Bioscience Co., Ltd, Zhuhai, Guangdong, China). PCR assays was performed according to the following protocol: 50 °C for 2 min, pre-denaturation at 95 °C for 15 min, followed by 45 cycles at 94 °C for 30 s and 65 °C for 45 s. The amplified products were revealed using an *APOE* Gene typing Detection kit (gene chip assay) (Sinochips Bioscience Co., Ltd, Zhuhai, China).

### Statistical analysis

Frequencies of the ∊2, ∊3 and ∊4 alleles were calculated by gene counting, e.g., the frequency of ∊2=(2* *APOE* ∊2/∊2 + *APOE* ∊2/∊3 + *APOE* ∊2/∊4)/ total number of alleles.

SPSS statistical software version 19.0 was used for data analysis. The data are reported as the means ± SD. Chi-square and Fisher’s exact tests were used to compare the allele and genotype frequencies. Descriptive analysis was used to compare allele frequencies between the Hakka population and published data of other ethnic groups. A value of *p* < 0.05 was considered as statistically significant.

### Results

**Figure 1:**
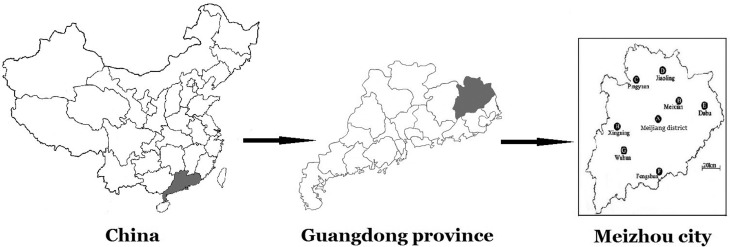
Geographical position of Meizhou in Guangdong Province of China.

A total of 6,907 subjects, 4,366 (63.21%) men and 2,541 (36.79%) women, were recruited in the study. The sample age ranged from 1 to 101 (64.06 ± 14.68) years, with means of 63.48 ± 14.62 in men and 65.06 ± 14.74 in women. Most of them came from southern China including seven areas of Meizhou city, Guangdong Province and some regions of Jiangxi Province, all of them are Hakka. The geographical position of Meizhou city is shown in Figure 1.

In this study, the genotype ∊3/∊3 accounted for 65.43% followed by ∊2/∊3 (15.85%), ∊3/∊4 (14.13%), ∊2/∊4 (3.01%), ∊4/∊4 (0.84%), and ∊2/∊2 (0.74%) in all subjects; ∊3 had the greatest allele frequency (80.42%) followed by ∊2 (10.17%) and ∊4 (9.41%). The results as showed in Table 1.

**Table 1:** Allele and genotype frequencies of *APOE* in 6907 participants in Hakka population.

*APOE*	Male (n=4366)			Female (n=2541)			Combined (n=6907)		
	n	Frequency	%	n	Frequency	%	n	Frequency	%
Allele									
∊2	899	0.103		506	0.100		1405	0.102	
∊3	7016	0.803		4093	0.805		11109	0.804	
∊4	817	0.094		483	0.095		1300	0.094	
Genotype									
∊2/∊2	29		0.66	22		0.87	51		0.74
∊2/∊3	710		16.26	385		15.15	1095		15.85
∊2/∊4	131		3.00	77		3.03	208		3.01
∊3/∊3	2851		65.30	1668		65.64	4519		65.43
∊3/∊4	604		13.83	372		14.64	976		14.13
∊4/∊4	41		0.94	17		0.67	58		0.84

## Discussion

ApoE is one of the important apolipoproteins in plasma, which is mainly synthesized, secreted, and metabolized in the liver (Schneider *et al.*, 1981; Rall Jr *et al.*, 1982b). It is involved in the transport, storage, and metabolism of lipids, and has the effects of repairing tissues, inhibiting platelet aggregation, and regulating immunity (van den Elzen *et al.*, 2005). Studies have found that *APOE* gene polymorphisms are closely associated with coronary heart disease, hyperlipidemia, cerebral infarction, Alzheimer’s disease, multiple sclerosis, chronic hepatitis, and other diseases (Ghiselli *et al.*, 1981; Corder *et al.*, 1993; Faivre *et al.*, 2005; Price *et al.*, 2006; Rovin *et al.*, 2007; Kathiresan *et al.*, 2008). ApoE4 is associated with decreased longevity, increased plasma total and LDL cholesterol, and increased prevalence of cardiovascular disease and Alzheimer’s disease. Different populations have different frequencies of genetic polymorphisms of *APOE* (Gerdes *et al.*, 1996).

In most populations, ∊3/∊3 is the commonest genotype while ∊3 is the commonest allele. In this study, genotype ∊3/∊3 accounted for 65.43% followed by ∊2/∊3 (15.85%), ∊3/∊4 (14.13%), ∊2/∊4 (3.01%), ∊4/∊4 (0.84%), and ∊2/∊2 (0.74%) in all subjects. ∊3 allele had the greatest allele frequency (80.42%) followed by ∊2 (10.17%) and ∊4 (9.41%). This was consistent with previous research on other populations.

We compared the allele frequencies estimated here for *APOE* ∊2, ∊3, and ∊4 allele with respect to previously published reports in other ethnic populations (Table 2). Comparison of our results with the geographically neighboring countries showed that the frequencies of ∊4 allele in Chinese populations were lower than in Javanese (Svobodova *et al.*, 2007a,b) populations, while the frequencies of the ∊2 allele were higher and of the ∊4 allele lower than in Japanese (Hallman *et al.*, 1991; Gerdes *et al.*, 1992) and Koreans (Hong *et al.*, 1997). In addition, the analysis showed that the frequencies of ∊2 and ∊4 allele in Hakka population were similar to the Vietnamese (Nghiem *et al.*, 2004), Chinese-Shanghai (Yang *et al.*, 2003), Chinese-Kunming Han (Tang *et al.*, 2005), Chinese-Northeast (Zhou *et al.*, 2005), and French (Boerwinkle *et al.*, 1986; Gueguen *et al.*, 1989; Bailleul *et al.*, 1993).

**Table 2:** Distribution of *APOE* (∊2, ∊3, ∊4) allele frequencies among major study populations.

Populations	Total Number	Alleles frequency of APOE			References
		∊2	∊3	∊4	
**Asians**					
Chinese					
Chinese-Hakka	6907	0.102	0.804	0.094	This work
Chinese-Shanghai	266	0.098	0.786	0.116	Yang *et al.,* 2003
Chinese-Dehong Dai	171	0.064	0.889	0.047	Tang *et al.,* 2005
Chinese- Jinangsu Han	168	0.071	0.863	0.066	Liang *et al.,* 2009
Chinese-Kunming Han	71	0.092	0.852	0.056	Tang *et al.,* 2005
Chinese-Northeast	69	0.096	0.824	0.081	Zhou *et al.,* 2005
Indian	4450	0.039	0.887	0.073	Thelma *et al.*, 2001
Japanese	1097	0.048	0.851	0.101	Hallmann *et al.,* 1991; Gerdes *et al.,* 1992
Mongolian	744	0.037	0.808	0.155	Svobodová *et al.,* 2007a
Vietnamese	348	0.090	0.790	0.120	Nghiem *et al.,* 2004
Malay	223	0.140	0.620	0.240	Gajra *et al.,* 1994a
Javanese	197	0.060	0.770	0.170	Gajra *et al.,* 1994b
Koreans	145	0.020	0.870	0.110	Hong *et al.,* 1997
Iranian	129	0.027	0.912	0.061	Raygani *et al.*, 2005
**Europeans**					
Dutch	2318	0.085	0.752	0.163	Smit *et al.*, 1988; Knjiff *et al.*, 1993
Finnish	2245	0.044	0.748	0.208	Lehtimäki *et al.*, 1990; Salo *et al.*, 1993; Hallman *et al.,* 1991
Germans	1211	0.083	0.784	0.133	Kolovou *et al.,* 2009
Italians	2000	0.060	0.849	0.091	Corbo *et al.,* 1995
Spanish	1286	0.052	0.856	0.091	Valveny *et al.*, 2010; Gerdes *et al.,* 1992; Lucotte *et al.,* 1997; Muros and Rodríguez-Ferrer*,* 1996
French	1228	0.108	0.771	0.121	Bailleul *et al.,* 1993; Gueguen *et al.,* 1989; Boerwinkle *et al.,* 1986
Belgians	189	0.069	0.762	0.169	Engelborghs *et al.*, 2003
UK	734	0.089	0.767	0.144	Corbo *et al.,* 1995; Lucotte *et al.,* 1997
Greeks	551	0.054	0.878	0.068	Marios *et al.*, 1995; Sklavounou *et al.*, 2010
Danish	466	0.085	0.741	0.174	Gerdes *et al.,* 1992
Swedish	407	0.077	0.740	0.190	Roussos *et al.*, 2004
Turks	90	0.063	0.868	0.069	Brega *et al.*, 1998
**Africans**					
Nigeria	1562	0.064	0.684	0.252	Kamboh *et al.*, 2015
Algerian	732	0.050	0.846	0.104	Boulenouar *et al.*, 2013
Sub-Saharans	470	0.116	0.706	0.178	Zekraoui *et al.*, 1997
Nigerians	365	0.027	0.677	0.296	Sepehrnia *et al.*, 1989
Khoi San	247	0.077	0.553	0.370	Sandholzer *et al.*, 1995
**North Americans**					
American- whites	702	0.082	0.778	0.140	Djoussé *et al.*, 2004
**South Americans**					
Brazil	2010	0.063	0.797	0.140	Fuzikawa *et al.*, 2007; França *et al.,* 2004; Brito *et al.*, 2011; Souza *et al.*, 2003
Venezuela	1841	0.055	0.834	0.111	Molero *et al.,* 2001; Arráiz *et al.*, 2010
Colombia	1001	0.075	0.814	0.111	Velez-Pardo *et al.*, 2015

**Figure 2:**
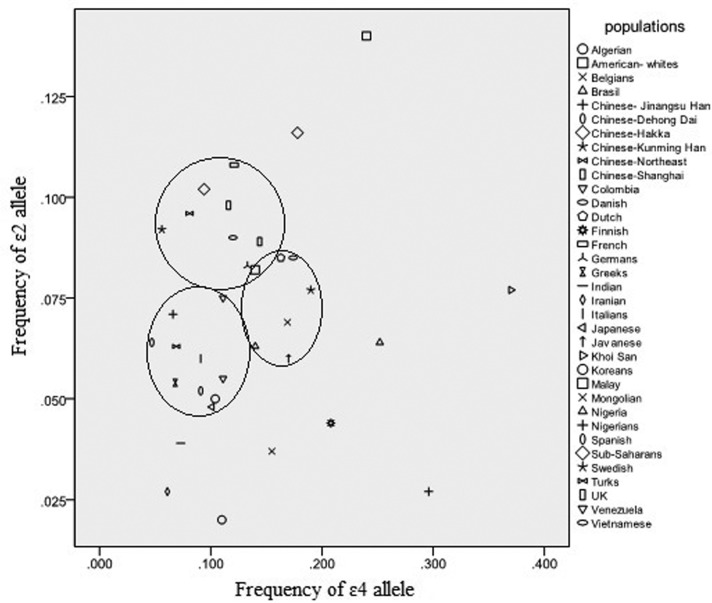
Distribution of *APOE* frequencies of ∊2 and ∊4 allele among major study populations.

Comparing our results with other Chinese populations, the frequencies of the ∊2 and ∊4 alleles in the Hakka population were highly similar to the Chinese-Shanghai, Chinese-Kunming Han, and Chinese-Northeast, while the frequency of ∊2 in the Hakka population was higher than Chinese-Dehong Dai (Tang *et al.*, 2005) and Chinese-Jiangsu Han (Liang *et al.*, 2009) (Figure 2). This suggests that the risk of some diseases in the Hakka population of Southern China may be different from those of other populations. Since ∊4 polymorphism is associated with increased risk of cardiovascular disease, Alzheimer’s disease, and other diseases, our findings suggest a low genetic risk in the Hakka population for these diseases.

In some reports, the subjects were relatively few and the results did not represent the actual gene frequencies of that region and population. Here, the Apolipoprotein E genetic polymorphism was analyzed in a large ethnic Hakka population in southern China, and is the first performed on a large sample of the population of this area. Our sample size is one of the largest of all studies, and thus should more accurately assess the *APOE* gene allele and genotype frequencies of the Hakka population in southern China. Our next step is to increase the sample size of the study. A number of investigations have demonstrated that carriers of ∊4 allele are characterized by a lower life expectancy (Hyman *et al.*, 1996; Gerdes *et al.*, 2015). Thus, we are going to investigate the *APOE* gene polymorphisms in people living in Jiaoling, which is considered the hometown of longevity in China.

## Conclusions

The frequencies of the ∊4 allele in Chinese populations were lower than in Mongolians and Javanese, while the frequencies of the ∊2 allele were higher and of the ∊4 allele lower than in Japanese and Koreans, which are geographically neighboring countries. The frequencies of the ∊2 and ∊4 alleles in the Hakka population were similar to the Vietnamese, Chinese-Shanghai, Chinese-Kunming Han and Chinese-Northeast, and French, while the frequency of ∊2 in the Hakka population was higher than Chinese-Dehong Dai and Chinese-Jinangsu Han. Our findings suggest a low genetic risk in the Hakka population for some diseases.
